# KI-gestützte EKG-Diagnostik: Warum klassische Teststatistik weiterhin entscheidend ist

**DOI:** 10.1007/s00399-026-01158-1

**Published:** 2026-06-23

**Authors:** Wilhelm Haverkamp, Gerhard Hindricks, Nils Strodthoff

**Affiliations:** 1 Klinik für Kardiologie, Angiologie und Intensivmedizin, Deutsches Herzzentrum der Charité (DHZC), Chariteplatz 1, 10117 Berlin, Deutschland; 2 Carls von Ossietzky Universität Oldenburg, Oldenburg, Deutschland

**Keywords:** Künstliche Intelligenz, Elektrokardiographie, Bayessches Theorem, Likelihood-Verhältnis, Überdiagnose, Artificial intelligence, Electrocardiography, Bayes’ theorem, Likelihood ratio, Overdiagnosis

## Abstract

Künstliche Intelligenz (KI) revolutioniert die Elektrokardiographie mit beeindruckenden Leistungskennzahlen. Doch diese Zahlen können irreführend sein, wenn klassische Konzepte der Teststatistik – insbesondere die Prätestwahrscheinlichkeit – nicht beachtet werden. Das Bayessche Theorem zeigt, dass selbst exzellente KI-Algorithmen bei niedriger Prävalenz zu einer Flut falsch-positiver Befunde führen können. Ärztinnen und Ärzte müssen daher die epidemiologischen Rahmenbedingungen ihrer Patientenkollektive kennen und KI-Ergebnisse stets kritisch im Kontext interpretieren. Die Zukunft liegt in der intelligenten Verbindung von KI und klinischem Urteil – nicht in der unreflektierten Übernahme algorithmischer Entscheidungen.

## Abstract (en)

Artificial intelligence (AI) is revolutionizing electrocardiography with impressive performance metrics. However, these figures can be misleading if classical test statistics—particularly pretest probability—are not considered. Bayes’ theorem demonstrates that even excellent AI algorithms can produce numerous false-positive results when disease prevalence is low. Likelihood ratios provide prevalence-independent assessment; calibration and clinical net benefit complement discrimination metrics such as area under the curve (AUC). Physicians must understand the epidemiological context of their patient populations and interpret AI results critically—not noncritically adopt algorithmic decisions.

Die moderne Medizin erlebt gegenwärtig eine tiefgreifende Transformation durch den Einsatz künstlicher Intelligenz (KI) , die insbesondere in der Elektrokardiographie (EKG) erhebliche Fortschritte erzielt [15, 23, 26]. KI-Algorithmen erkennen heute aus dem 12-Kanal-EKG nicht nur klassische Diagnosen, sondern auch eine reduzierte linksventrikuläre Funktion [3], hypertrophe Kardiomyopathie [17], kardiale Amyloidose [12] sowie Hyperkaliämie [10]; weitere Anwendungen reichen bis zur prädiktiven Erkennung künftiger Rhythmusstörungen vor deren klinischer Manifestation [2, 27]. Begleitend werden Leistungskennzahlen präsentiert, die außergewöhnlich wirken: hohe Sensitivität und Spezifität sowie Flächen unter der ROC-Kurve (AUC), die als „sehr gut“ bis „exzellent“ gelten [26].

Diese eindrucksvollen Zahlen erzeugen hohe Erwartungen, die jedoch trügen können. Denn die tatsächliche Aussagekraft eines diagnostischen Tests wird nicht allein durch seine technischen Kennwerte bestimmt, sondern wesentlich durch die Prätestwahrscheinlichkeit der Zielerkrankung in der jeweils untersuchten Population [13, 24]. Das Bayessche Theorem als grundlegendes Prinzip der Wahrscheinlichkeitstheorie [4] zeigt dabei, dass selbst leistungsstarke Tests bei seltenen Ereignissen eine große Zahl falsch-positiver Ergebnisse erzeugen können – mit potenziell weitreichenden klinischen, psychologischen und ökonomischen Konsequenzen [6, 20]. Die jüngst publizierte EHRA/HRS-Stellungnahme zur KI in der klinischen Elektrophysiologie unterstreicht die Bedeutung einer sachgerechten Berichterstattung und Bewertung von KI-Studien und stellt mit der EHRA-AI-Checkliste einen entsprechenden Rahmen bereit [27].

## Grundbegriffe der Teststatistik

### Sensitivität (Sens):

Anteil der korrekt als krank erkannten Personen unter allen tatsächlich Kranken. Beantwortet die Frage: Wenn jemand krank ist, wie wahrscheinlich ist der Test positiv?

### Spezifität (Spez):

Anteil der korrekt als gesund erkannten Personen unter allen tatsächlich Gesunden. Beantwortet die Frage: Wenn jemand gesund ist, wie wahrscheinlich ist der Test negativ?

### Prävalenz:

Häufigkeit der Erkrankung in der untersuchten Population (= Prätestwahrscheinlichkeit bei asymptomatischen Personen).

### Prätestwahrscheinlichkeit:

Wahrscheinlichkeit, dass eine Person die Zielerkrankung hat, *bevor* der diagnostische Test angewandt wird (in Screening-Situationen entspricht sie der Prävalenz).

### Posttestwahrscheinlichkeit:

Aktualisierte Wahrscheinlichkeit für das Vorliegen der Erkrankung *nach* Vorliegen des Testergebnisses. Bei positivem Test entspricht sie dem positiv-prädiktiven Wert (PPV); bei negativem Test ist sie 1 minus dem negativ-prädiktiven Wert (NPV).

### PPV (positiv-prädiktiver Wert):

Wahrscheinlichkeit, bei positivem Test tatsächlich erkrankt zu sein. Beantwortet die Frage: Mein Test ist positiv – wie wahrscheinlich bin ich wirklich krank?

### NPV (negativ-prädiktiver Wert):

Wahrscheinlichkeit, bei negativem Test tatsächlich gesund zu sein. Beantwortet die Frage: Mein Test ist negativ – wie wahrscheinlich bin ich wirklich gesund?

Im Folgenden zeigen wir, warum klassische Teststatistiken wie Sensitivität, Spezifität, ROC/AUC, Likelihood-Verhältnisse, Prä- und Posttestwahrscheinlichkeiten auch im KI-Zeitalter unverzichtbar sind. Als Beispiel dient eine prominente Arbeit zur Vorhersage von Vorhofflimmern aus Sinusrhythmus-EKGs [2]; die Prinzipien sind jedoch allgemeingültig und auf andere KI-Diagnostik-Anwendungen übertragbar.

## Die Verlockung der Perfektion: wie Standard-Metriken täuschen können

Die Bewertung diagnostischer KI-Algorithmen folgt etablierten statistischen Maßstäben [5, 27]. Im Fokus stehen Sensitivität (Erkrankte korrekt erkennen) und Spezifität (Gesunde korrekt ausschließen) [1]. Hohe Werte suggerieren Sicherheit; eine Fläche unter der ROC-Kurve (AUC) *≥ 0,9* wird klassisch als „ausgezeichnet“ eingeordnet [14]. Diese Schwelle ist allerdings kontextabhängig: Eine AUC von 0,90 in einer kuratierten Studie kann in einer realen Versorgungssituation mit anderem Spektrum, anderer Erkrankungshäufigkeit und anderer Datenqualität deutlich niedriger ausfallen. Die AUC misst zudem ausschließlich die Diskrimination und sagt nichts über die *Kalibration* aus, also darüber, ob die ausgegebenen Wahrscheinlichkeiten den tatsächlichen Erkrankungsraten entsprechen [29] – ein Modell kann hervorragend diskriminieren und gleichzeitig systematisch über- oder unterschätzen. Die genannten Metriken entstehen zudem häufig unter idealisierten Studienbedingungen mit kuratierten Datensätzen, künstlich angereicherten Fallzahlen und retrospektiv gesicherten Endpunkten; die klinische Realität ist unordentlicher und weist oft niedrigere Prävalenzen auf [7]. Eine vollständige Modellbewertung sollte daher neben Diskrimination und Kalibration auch den klinischen Nettonutzen prüfen, etwa mittels Decision-Curve-Analyse [30].

Konsequenz: Ein Test, der im Paper exzellent wirkt, kann im Alltag – bei anderer Prätestwahrscheinlichkeit – zahlreiche falsch-positive Ergebnisse produzieren. Die klassische Teststatistik liefert das Rüstzeug, um diese Diskrepanz zu verstehen und zu kommunizieren.

Im Vergleich zum menschlichen Befunder erreichen tiefe neuronale Netze in der Routine-EKG-Klassifikation eine Trefferquote, die mit erfahrenen Kardiologen vergleichbar ist und die unerfahrener Befunder häufig übertrifft [15, 23]. Bei subklinischen, dem Auge weitgehend verborgenen Mustern – etwa der Detektion einer reduzierten linksventrikulären Funktion [3] oder einer AF-Disposition [2] – können sie den menschlichen Befunder sogar systematisch übertreffen. Dies hebt die teststatistischen Fallstricke jedoch nicht auf: Auch eine im Vergleich „bessere“ KI hat bei niedriger Prätestwahrscheinlichkeit einen geringen PPV und erfordert vor therapeutischen Konsequenzen eine Bestätigungsdiagnostik [21].

## Exemplarischer Anwendungsfall: Vorhersage von Vorhofflimmern bei Sinusrhythmus im EKG

Die Studie von Attia et al. evaluierte einen KI-Algorithmus, der aus einem normalen Sinusrhythmus-EKG eine Disposition für das spätere Auftreten von Vorhofflimmern vorhersagen sollte [2]. In einem Kollektiv von über 180.000 Patienten mit über 649.000 EKGs lag die AUC bei 0,87; berichtet wurden eine Sensitivität von 79 % und eine Spezifität von 79,5 %.

Die Studienprävalenz der Zielarrhythmie lag bei 8,4 % [2] – eine durch das retrospektive Studiendesign mit gezielter Patientenselektion angereicherte Kohorte („enriched population“), typisch für deutlich ältere, teils multimorbide Patienten [8]. Diese Anreicherung ist methodisch sinnvoll für die Entwicklung von Algorithmen, spiegelt aber nicht die klinische Realität in vielen Versorgungssituationen wider. Unter Studienbedingungen erscheint die Testleistung vielversprechend und der Test scheint oberflächlich betrachtet klinisch einsetzbar.

**Abb. 1:**
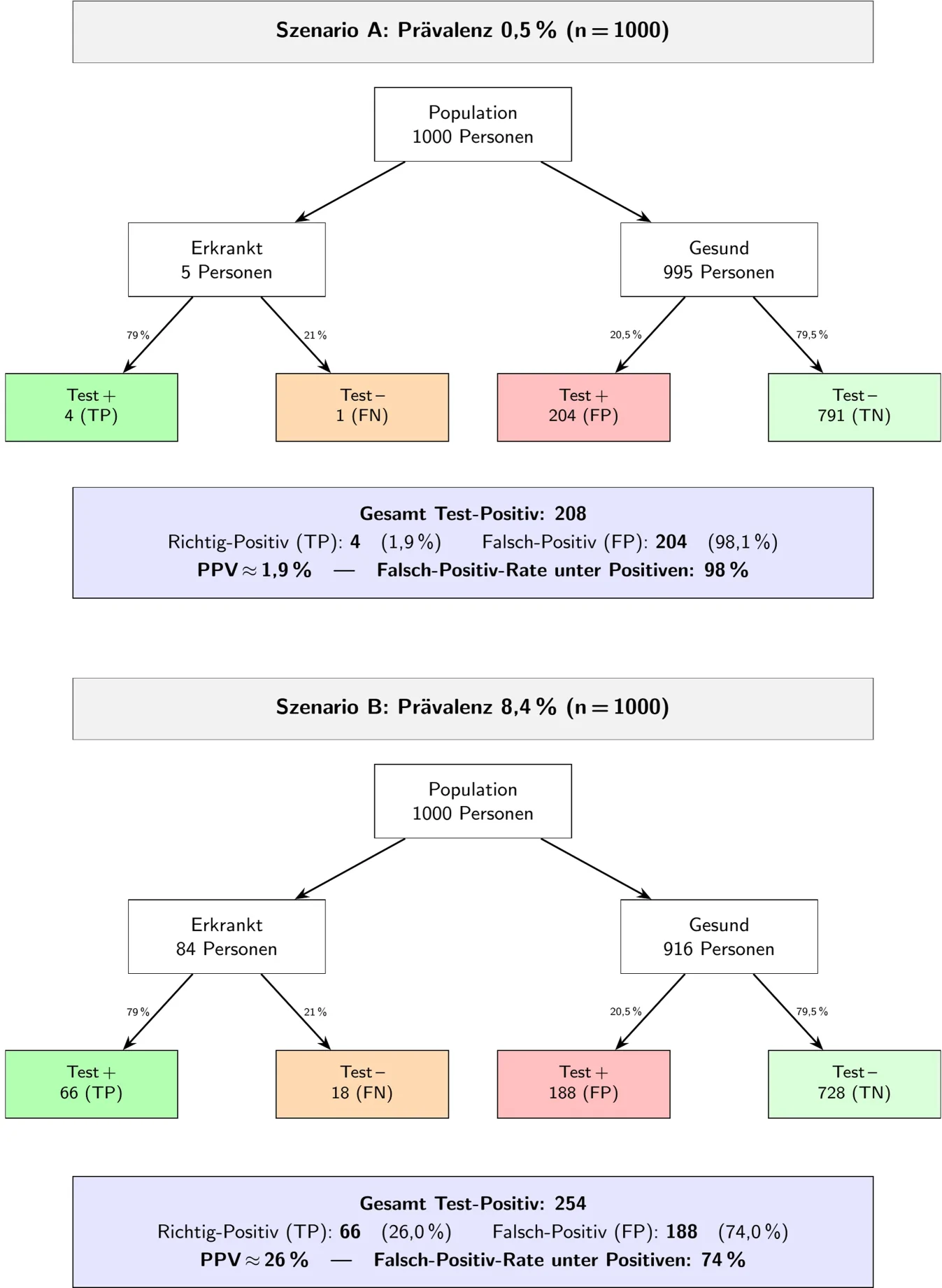
*Bayes-Effekt: Einfluss der Prävalenz auf die Testleistung eines KI-Algorithmus zur Vorhersage von Vorhofflimmern* (Sensitivität 79 %, Spezifität 79,5 % nach Attia et al. [1]). *Szenario* *A (oben):* Bei niedriger Prävalenz (0,5 %, entsprechend einer 50-jährigen Allgemeinbevölkerung) sind 98 % der positiven Testergebnisse falsch-positiv (PPV 1,9 %). *Szenario* *B (unten):* Bei höherer Prävalenz (8,4 %, Studienpopulation) sind noch 74 % der positiven Testergebnisse falsch-positiv (PPV 26 %). Die identische Testcharakteristik führt zu dramatisch unterschiedlicher klinischer Aussagekraft. *TP* richtig-posit*iv*, *FP* falsch-positiv, *TN* richtig-negativ, *FN* falsch-negativ, *PPV* positiv-prädiktiver Wert

Doch was geschieht in einer Population mit niedriger Prävalenz? Wenn wir dieselben Testcharakteristika auf 50-Jährige mit einer Prävalenz von ca. 0,5 % übertragen [11], verschiebt sich die Aussagekraft dramatisch – ein klassischer Bayes-Effekt (Abb. 1).

### Wie „sieht“ die KI das EKG?

Modernen KI-EKG-Algorithmen liegen meist tiefe neuronale Netze (Convolutional Neural Networks) zugrunde, die direkt auf der digitalen Rohspur des 12-Kanal-EKGs arbeiten und – anders als der menschliche Befunder – nicht einzelne Wellen, sondern hierarchische Mustermerkmale lernen [15, 26]. Welche EKG-Regionen für die Entscheidung des Netzes besonders bedeutsam sind, lässt sich mit Verfahren der erklärbaren KI sichtbar machen, etwa Saliency- oder Class-Activation-Maps [27]. Bei Algorithmen zur Detektion einer reduzierten linksventrikulären Funktion oder einer AF-Disposition fokussieren diese Karten typischerweise nicht auf eine einzelne Auffälligkeit, sondern auf subtile, ableitungsübergreifende Änderungen der P‑Welle, der QRS-Morphologie und der Repolarisation – Muster, die für das menschliche Auge im Sinusrhythmus oft nicht erkennbar sind.

### Das Bayessche Theorem in der Praxis

Das Bayessche Theorem quantifiziert die Beziehung zwischen Prätestwahrscheinlichkeit, Testgüte und Posttestwahrscheinlichkeit [4, 24]:

$$\mathrm{PPV}= \frac{\mathrm{Sens}\times \text{Pr{\"a}valenz}}{\begin{array}{c}\mathrm{Sens}\times \text{Pr{\"a}valenz}\\{}+\left(1-\mathrm{Spez}\right)\\{}\times \left(1-\text{Pr{\"a}valenz}\right)\end{array}}$$

Diese Formel zeigt: Der PPV hängt nicht nur von Sensitivität und Spezifität ab, sondern entscheidend von der Prävalenz. Bei niedriger Prävalenz dominiert der Term $\left(1-\mathrm{Spez}\right)\times \left(1-\text{Pr{\"a}valenz}\right)$ im Nenner, wodurch der PPV klein wird – selbst bei guter Testleistung.

### Niedrige Prävalenz von Vorhofflimmern (0,5 %, Population der 50-Jährigen)

Für die in der Studie nachgewiesene Sensitivität von 79 % und Spezifität von 79,5 % ergibt sich bei einer Prävalenz von 0,5 %:

$$\begin{aligned}\mathrm{PPV}&=\frac{0,79\times 0,005}{0,79\times 0,005+0,205\times 0,995}\\{}&=\frac{0,00395}{0,00395+0,204}\approx 0,019\\{}&=1,9\mathrm{{\%}}\end{aligned}$$

Bei einer Modellrechnung mit 1000 50-jährigen Patienten ergibt sich, wie in Abb. 1 (Szenario A) dargestellt, Folgendes:

- Es gibt 5 tatsächlich Erkrankte (0,5 %), davon sind 4 richtig-positiv (79 %) und einer falsch-negativ.
- Es gibt 995 Gesunde, davon sind 791 korrekt negativ (79,5 % von 995) und 204 falsch-positiv (20,5 % von 995).
- Insgesamt ergeben sich 208 positive Tests (4 + 204), davon sind nur vier korrekt. PPV = 4/208 *≈* 1,9 %.
- *→* Falsch-positive Rate unter den Positiven: 204/208 *≈* 98 %.

#### Klinische Interpretation:

Von 100 positiv getesteten 50-Jährigen haben nur etwa 2 tatsächlich Vorhofflimmern – 98 sind falsch-positive Befunde.

### Höhere Prävalenz von Vorhofflimmern (8,4 %, Studienpopulation)

Bei einer Prävalenz von 8,4 % wären unter 1000 getesteten Personen 84 tatsächlich erkrankt und 916 gesund. Abb. 1 (Szenario B) zeigt, dass der Test *≈* 66 richtig-positive Ergebnisse (79 % von 84) und *≈* 18 falsch-negative Ergebnisse liefern würde. Von den gesunden Personen erhielten *≈* 188 (20,5 % von 916) ein falsch-positives Resultat, 728 würden korrekt ausgeschlossen. Somit ergäben sich insgesamt 254 positive Tests (66 + 188), von denen 188 (*≈* 74 %) falsch-positiv wären. Der PPV steigt auf *≈* 26 %, der NPV auf *≈* 98 %.

#### Klinische Interpretation:

Selbst in dieser angereicherten Studienpopulation wären noch 3 von 4 positiven Tests falsch-positiv.

Diese Betrachtung zeigt: Auch bei identischer Testcharakteristik hängt der klinische Wert eines positiven Tests entscheidend von der Prävalenz ab.

### Likelihood-Verhältnisse: prävalenzunabhängige Orientierung

In der klinischen Praxis stellt sich die Frage, wie Testcharakteristika unabhängig von konkreten Prävalenzen bewertet werden können. Genau hier setzen die Likelihood-Verhältnisse (LR) an [9, 19]: Das LR beschreibt, um welchen Faktor sich die Wahrscheinlichkeit für das Vorliegen einer Erkrankung durch ein Testergebnis ändert. Konkret werden mit Hilfe der LR die *Prätestodds* (= Prätestwahrscheinlichkeit/[1 − Prätestwahrscheinlichkeit]) in *Posttestodds* überführt: Posttestodds = Prätestodds × LR. Über die Beziehung Wahrscheinlichkeit = Odds/(1 + Odds) lässt sich daraus die Posttestwahrscheinlichkeit für jede Prätestwahrscheinlichkeit berechnen [9, 19].

#### Likelihood-Verhältnisse (LR)

##### LR+ (positives Likelihood-Verhältnis):

Um welchen Faktor steigt die Wahrscheinlichkeit bei positivem Test?

- LR+ *> 10*: Starker Hinweis auf Erkrankung
- LR+ 5–10: Moderater Hinweis
- LR+ 2–5: Schwacher Hinweis
- LR+ *< 2*: Kaum aussagekräftig

##### LR− (negatives Likelihood-Verhältnis):

Um welchen Faktor sinkt die Wahrscheinlichkeit bei negativem Test?

- LR− *< 0,1*: Erkrankung sehr unwahrscheinlich
- LR− 0,1–0,2: Erkrankung unwahrscheinlich
- LR− 0,2–0,5: Mäßiger Ausschluss
- LR− *> 0,5*: Schwacher Ausschluss

#### Positives Likelihood-Verhältnis (LR+)

$$\vrule height6mm width0mm \mathrm{LR}+=\frac{\text{Sensitivit{\"a}t}}{1-\text{Spezifit{\"a}t}}=\frac{0,79}{0,205}=3,85$$

Dieser Wert ist ein schwacher bis moderater Hinweis auf Erkrankung; ein positiver Test allein reicht nicht für eine definitive Diagnose.

#### Negatives Likelihood-Verhältnis (LR−)

$$\vrule height6mm width0mm \mathrm{LR}-=\frac{1-\text{Sensitivit{\"a}t}}{\text{Spezifit{\"a}t}}=\frac{0,21}{0,795}=0,26$$

Dieser Wert spricht für einen mäßigen Ausschluss: Ein negativer Test reduziert das Risiko deutlich.

### Interpretation

Bei einer Prävalenz von 0,5 % sinkt die Posttestwahrscheinlichkeit nach negativem Test auf *≈* 0,13 % – beruhigend für die klinische Praxis. Ein positiver Test erhöht das Risiko nur auf *≈* 1,9 %.

Bei einer Prävalenz von 8,4 % steigt die Posttestwahrscheinlichkeit nach positivem Test auf *≈* 26 %; nach negativem Test sinkt sie auf *≈* 2 %.

#### Fazit:

Der Test eignet sich deutlich besser zum Ausschluss als zum sicheren Nachweis der Erkrankung. Die klinische Interpretation muss stets die Prävalenz der Zielpopulation berücksichtigen.

## Klinische Konsequenzen

Die statistischen Einsichten haben direkte praxisrelevante Folgen. Ein positives KI-Screening in einer Niedrigprävalenz-Population darf nicht als Diagnose gewertet werden. Wichtig ist eine begriffliche Klarstellung: Aktuelle Leitlinien zur Antikoagulation bei Vorhofflimmern verlangen ausdrücklich die EKG-Dokumentation der Rhythmusstörung als Voraussetzung für eine Therapieeinleitung – eine bloße KI-basierte Risikoprädiktion aus dem Sinusrhythmus-EKG ist hierfür nicht ausreichend [16]. Die Gefahr besteht deshalb weniger in einer direkten Therapieeinleitung als in den nachgelagerten Konsequenzen: Ein bei einem 50-Jährigen mit einer Posttestwahrscheinlichkeit von ca. 2 % gestellter „KI-positiver“ Befund kann unkritisch als „beginnendes Vorhofflimmern“ interpretiert werden, weitere – teils invasive – Diagnostik nach sich ziehen, die psychische Belastung erhöhen und den Behandlungspfad in Richtung prophylaktischer Therapie verschieben [6, 20]. Damit kann ein methodisch korrekt entwickelter Algorithmus indirekt Schaden anrichten, wenn er außerhalb seines validierten Einsatzkontextes interpretiert wird.

Sinnvoll ist deshalb ein gestuftes Vorgehen: Ein positives KI-Screening sollte zielgerichtetes Monitoring (verlängerte EKG-Registrierung, Ereignisrekorder, Wearables) nach sich ziehen [25], um – falls vorhanden – den eigentlichen Rhythmusbefund zu sichern, der dann gemäß Leitlinie therapierelevant ist [16]. Auch psychologische Auswirkungen falsch-positiver Befunde sind zu berücksichtigen: Angst, Verunsicherung, „Patientifizierung“ trotz niedriger tatsächlicher Wahrscheinlichkeit [6, 20] – mit individuellen und ökonomischen Folgen.

## Strategien für den verantwortungsvollen Umgang mit KI-Diagnostik

Die Erfahrungen mit KI-gestützten EKG-Algorithmen verdeutlichen, dass diagnostische Innovationen nur dann klinisch nutzbringend eingesetzt werden können, wenn sie von einem reflektierten, statistisch fundierten Vorgehen begleitet werden.

### Grundprinzipien

Es gibt mehrere wichtige Grundprinzipien, derer sich Anwender auch bei der KI-gestützten Diagnostik bewusst sein sollten:

1. **Epidemiologie kennen.** Die Aussagekraft eines Tests hängt wesentlich von der Prävalenz der Zielerkrankung in der untersuchten Population ab. Ärztinnen und Ärzte sollten daher die epidemiologischen Verhältnisse in ihrem eigenen Umfeld kennen.
2. **Likelihood-Verhältnisse nutzen.** Während Sensitivität, Spezifität und AUC wichtige Kennzahlen sind, geben sie in der klinischen Praxis oft nur ein unvollständiges Bild. Likelihood-Verhältnisse erlauben eine prävalenzunabhängige Bewertung; Kalibration und Decision-Curve-Analyse ergänzen den klinischen Nettonutzen [29, 30].
3. **Kontext-sensitives Thresholding.** In einem Screening-Szenario mit niedriger Prävalenz ist es sinnvoll, die Spezifität zu priorisieren, um die Zahl falsch-positiver Ergebnisse zu reduzieren.
4. **Gestufte Diagnostik.** KI-Screening sollte als Einstieg in einen diagnostischen Prozess verstanden werden. Ein positives Ergebnis sollte grundsätzlich durch spezifischere Methoden bestätigt werden, bevor therapeutische Konsequenzen gezogen werden.
5. **Transparente Kommunikation.** Es ist essenziell, klar zu vermitteln, dass ein positives Ergebnis keine definitive Diagnose bedeutet, sondern eine statistische Risikoeinschätzung.
6. **Multimodale Integration.** Anamnese, klinische Untersuchung, Laborwerte, Bildgebung oder Daten aus Wearables können die Prätestwahrscheinlichkeit erhöhen und so die Aussagekraft verbessern.
7. **Evaluation in Zielpopulationen.** EHRA-AI-Checkliste, CONSORT-AI und TRIPOD-AI sollten konsequent angewandt werden; KI-Algorithmen müssen in den realen Zielgruppen prospektiv validiert werden [18, 27].

### Klinisches Beispiel: Hausarztpraxis versus Rhythmuszentrum

Die Vorteile kontextsensitiver KI lassen sich gut an zwei kontrastierenden Szenarien verdeutlichen:

#### Hausarztpraxis, 50-jährige Population (niedrige Prävalenz *≈* 0,5 %):

Hier führt der Einsatz eines KI-Screenings mit fixem Schwellenwert zu einer Flut falsch-positiver Ergebnisse. Adaptive Algorithmen könnten in diesem Setting die Spezifität erhöhen, indem sie höhere Entscheidungsschwellen anlegen.

#### Universitäres Rhythmuszentrum, ältere multimorbide Patienten (hohe Prävalenz $> 10\mathrm{{\%}}$):

In dieser Population ist die Wahrscheinlichkeit einer Arrhythmie deutlich höher. Hier wäre es sinnvoll, die Sensitivität zu priorisieren, um möglichst wenige Erkrankte zu übersehen.

## Die Zukunft der KI-Diagnostik: Präzision durch Kontext

Die nächsten Entwicklungsschritte der KI-Diagnostik werden nicht primär in der weiteren Steigerung isolierter testbezogener Leistungskennzahlen liegen, sondern in der Kontextualisierung. Adaptive Algorithmen könnten in Zukunft Entscheidungsschwellen dynamisch justieren, abhängig vom individuellen Risikoprofil des Patienten. Ein weiterer wichtiger Schritt ist die multimodale Fusion, bei der EKG-Daten mit zusätzlichen Informationsquellen verknüpft werden.

Gleichzeitig bleibt eine fundamentale Einsicht bestehen: KI kann die klassische Teststatistik nicht ersetzen, sondern baut auf ihr auf. Auch die modernsten adaptiven oder multimodalen Verfahren müssen im Lichte von Sensitivität, Spezifität, Prävalenz und Bayesschem Theorem interpretiert werden.

## Schlussfolgerung

KI kann die Gesetze der Wahrscheinlichkeit nicht suspendieren. Das Bayessche Theorem bleibt auch im Zeitalter des maschinellen Lernens der Maßstab für die klinische Aussagekraft diagnostischer Tests [4]. Wer KI-Ergebnisse ohne Kontext interpretiert, riskiert Überdiagnose und Fehlsteuerung.

Die intelligente Integration von KI-Modellen mit klassischer Teststatistik und ärztlicher Urteilskraft ist der Weg, das Potenzial der Technologie verantwortungsvoll zu heben. Ärztinnen und Ärzte bleiben die entscheidenden Interpreten und Gatekeeper. Nur so lassen sich Patientennutzen maximieren und Fallstricke vermeiden [22, 28].

## Offene Fragen

Mehrere Fragen bleiben offen: Wie ist der prospektive klinische Nutzen KI-basierter Risikoprädiktion außerhalb hochangereicherter Studienkohorten? Wie lässt sich die Kalibration über heterogene Versorgungsregionen hinweg sichern? Welche regulatorischen Rahmenbedingungen sind für ein dynamisches Threshold-Tuning erforderlich? Und wie können Wearable-basierte und arztzentrierte Datenströme so verknüpft werden, dass die Prätestwahrscheinlichkeit individuell präzisiert wird – ohne Überdiagnose zu erzeugen?

## Fazit für die Praxis

Die Prätestwahrscheinlichkeit ist entscheidend: Auch ein KI-EKG-Algorithmus mit AUC >0,85 kann in Niedrigprävalenz-Populationen > 95 % falsch-positive Befunde produzieren. Die AUC ist dabei nicht alles – Diskrimination, Kalibration und klinischer Nettonutzen (z. B. Decision-Curve-Analyse) gehören gemeinsam zur vollständigen Modellbewertung. Statt sich auf den positiv-prädiktiven Wert zu verlassen, erlauben Likelihood-Verhältnisse eine prävalenzunabhängige Übersetzung des Testergebnisses in die individuelle Posttestwahrscheinlichkeit. Ein KI-Befund ist keine Diagnose: Therapierelevante Entscheidungen – etwa eine Antikoagulation bei Vorhofflimmern – verlangen weiterhin die EKG-Dokumentation der Arrhythmie. Sinnvoll ist daher eine gestufte Diagnostik, bei der ein positiver KI-Screening-Befund zu zielgerichtetem Monitoring führt, nicht zu unmittelbarer Therapie. Reporting-Standards wie die EHRA-AI-Checkliste, CONSORT-AI und TRIPOD-AI bilden den Rahmen für eine sachgerechte Bewertung publizierter Modelle.

## Data Availability

Dieser Artikel basiert auf bereits veröffentlichten Daten. Keine neuen Datensätze wurden generiert oder analysiert.
